# Addressing implicit bias in pharmacy: strategies for equitable patient care and improved medication outcomes

**DOI:** 10.3389/fpubh.2026.1763446

**Published:** 2026-04-02

**Authors:** Fahad Alzahrani, Faris S. Alnezary

**Affiliations:** Department of Pharmacy Practice, College of Pharmacy, Taibah University, Madinah, Saudi Arabia

**Keywords:** health disparities, health equity, implicit bias, patient-centered care, pharmaceutical care, pharmacists

## Abstract

**Objective:**

As pharmacists’ roles expand to include comprehensive clinical care, the quality of the pharmacist-patient relationship is increasingly critical to health outcomes. This narrative review examines the impact of implicit bias—the unconscious attitudes and stereotypes that influence perception and action on equitable pharmacy practice and synthesizes evidence-based strategies to mitigate its effects.

**Findings:**

A growing body of evidence confirms that implicit bias is a tangible factor in pharmacy practice, manifesting through disparities in communication, patient assessment, and clinical decision-making. While these biases are malleable through deliberate intervention, current research is limited by small-scale designs and a lack of pharmacy-specific measurement tools. Furthermore, the impact of bias-reduction on long-term clinical outcomes and the potential of emerging technologies, such as artificial intelligence, remain largely underexplored.

**Summary:**

This work presents a multi-level framework to address implicit bias in the pharmacy setting. At the individual level, strategies such as structured self-reflection (e.g., using the Implicit Association Test), perspective-taking, and mindfulness are essential to build practitioner awareness. At the organizational level, systemic changes such as standardizing care processes, fostering an inclusive environment, and implementing robust training programs are necessary to support individual efforts and ensure sustained improvement. Furthermore, integrating bias mitigation and cultural humility into pharmacy education curricula is fundamental in preparing the next generation of practitioners. Addressing implicit bias is not merely an ethical imperative but is, rather, a core component of high-quality, patient-centered care. A dedicated commitment to these strategies is crucial for advancing health equity and fulfilling the evolving role of the pharmacist.

## Introduction

Over the past several decades, the profession of pharmacy has gradually expanded beyond traditional dispensing responsibilities toward a broader, patient-centered clinical role (1). Today, pharmacists are integral members of the healthcare team, providing services such as Medication Therapy Management (MTM), chronic disease state management, immunizations, and preventative health screenings (2, 3). This evolution places the pharmacist in a position of significant influence over patient health outcomes, making the quality of the pharmacist-patient therapeutic alliance more critical than ever. However, the potential of this expanded role is fundamentally undermined by the persistence of health disparities, many of which are perpetuated by implicit biases, unconscious attitudes, or stereotypes that can influence decision-making and patient interactions (4–6).

Evidence shows that implicit bias exists among pharmacy professionals and students, manifesting in subtle differences in communication, empathy, and willingness to provide services, which can, in turn, contribute to inequitable care and poorer medication outcomes for marginalized populations (4, 7, 8).

This review examines how implicit bias contributes to these disparities within pharmacy practice and synthesizes a framework of evidence-based strategies that pharmacists can use to identify and mitigate their own biases. While much of the foundational research in this area originates from North America, this issue has global relevance, and the strategies presented herein are broadly applicable.

As the pharmacy profession continues to advance, addressing implicit bias through targeted education, reflective practice, and organizational change is essential to ensure equitable patient care and optimize medication outcomes for all individuals (6, 9, 10).

## Disparities in healthcare

Healthcare disparities and health inequities are related but distinct concepts. Health disparities (or health differences) refer to any quantifiable difference in health status, access to care, or outcomes between socially, economically, demographically, or geographically defined groups (11). In contrast, health inequities are a subset of these disparities that are systematic, potentially remediable, and specifically deemed to be unjust or unfair (12). These inequities often arise from differences in the quality, appropriateness, and continuity of care, rather than just income or insurance status. Evidence from diverse settings demonstrates that marginalized groups, including racial and ethnic minorities, as well as individuals disadvantaged by age, sexual orientation, disability, or stigmatized conditions, frequently encounter these avoidable barriers to equitable treatment (13, 90, 91). Such inequities represent a significant obstacle to fairness and optimal health outcomes within global health systems (14).

These inequities manifest across the continuum of care, including delayed access to services, variations in treatment intensity and quality, and disparities in health outcomes. Within the realm of pharmacy practice, for example, this can translate into marginalized patients who are less likely to be offered advanced services such as MTM, who receive less thorough counseling on complex medications, including inhalers and biologics, and who face heightened scrutiny when filling legitimate prescriptions for controlled substances (8, 15, 16). The impact is particularly pronounced in chronic disease management, where sustained and equitable care is essential in preventing complications, reducing hospitalizations, and improving long-term prognoses (16, 17). The roots of these disparities are complex and multifactorial, involving the interaction of systemic bias and organizational barriers with the social determinants of health (SDoH), the conditions in which people are born, live, work, and age, such as income, education, and neighborhood environment, that shape access to resources and health outcomes (18–20). Underfunded institutions, fragmented models of care, and limited availability of linguistically and culturally appropriate services further exacerbate the problem (21–23). For patients with chronic conditions, these challenges often lead to delayed diagnoses, diminished continuity of care, and difficulties navigating treatment plans, ultimately widening existing health gaps (24).

Resource constraints and workforce limitations also perpetuate inequities. Underserved communities commonly face shortages of healthcare providers and pharmacies, limited access to preventive services, and fewer opportunities for patient education. High patient volumes, relentless time pressures, and insufficient cultural competence training further hinder providers’ ability to deliver equitable care. Although targeted reforms—such as expanded insurance coverage and community health initiatives—have improved access in certain areas, progress remains inconsistent (25–27). Achieving sustainable health equity will require multilevel strategies that simultaneously address systemic challenges in healthcare delivery and the broader social determinants of health.

## The nature of implicit bias

Implicit biases begin forming early in life through socialization and repeated exposure to cultural norms, media portrayals, and societal stereotypes, shaping automatic associations that operate outside conscious awareness (28–30). Research in implicit social cognition suggests that these biases reflect patterns and inequalities present in an individual’s environment rather than innate prejudices (31). Young children acquire racial and other group biases “from mere observation” of others’ behavior and nonverbal signals, with implicit racial biases detectable as early as preschool and remaining relatively stable across childhood (32). Research shows that children acquire racial and other group biases through observation of social cues, with implicit biases detectable as early as preschool and remaining relatively stable across development (29). These findings highlight how repeated experiences and cultural influences contribute to the formation of implicit stereotypes (33, 34).

In clinical settings, high workloads, frequent interruptions, and diagnostic uncertainty increase reliance on automatic thinking, making implicit bias more likely to influence decision-making (35, 36). Although often conflated, implicit bias and stigma are distinct yet synergistic. Implicit bias involves the unconscious associations that shape perception and choice, while stigma represents the socially constructed negative labels or stereotypes assigned to specific groups.

Stigma acts as a catalyst, reinforcing and activating these latent biases during patient encounters (37, 38). Specific patient-related cues, including physical appearance, accents, demeanor, or requests for mental health and controlled medications, can trigger these stigmatizing frameworks. This dynamic contributes to documented disparities in diagnosis, treatment quality, and patient engagement, particularly for minority populations and other marginalized groups (35, 36, 39–41).

## Implicit bias in healthcare

A growing body of research identifies implicit bias among healthcare professionals as a critical factor contributing to these persistent disparities. Implicit biases are unconscious attitudes or stereotypes that affect our understanding, actions, and decisions (4). Neuroscientifically, these biases are rooted in the brain’s dual-process architecture, specifically the tension between “System 1” and “System 2” thinking (42).

System 1 is an evolutionary adaptation that allows for fast, automatic, and intuitive processing using heuristics, mental shortcuts that help the brain manage the vast amount of information it receives daily (43). While these shortcuts are necessary for rapid decision-making, they are frequently shaped by societal stereotypes and cultural norms, leading to the formation of unconscious associations (30). Because these processes often occur automatically, they can bypass our conscious, egalitarian “System 2” values, explaining why even well-intentioned healthcare professionals still demonstrate implicit preferences (44).

In contrast, System 2 is slow, deliberate, and analytical (42). In healthcare, tensions often arise between explicit egalitarian values (System 2) and these automatic, stereotype-driven responses (System 1) (4). Because high-pressure clinical settings increase reliance on System 1 thinking, implicit bias can become more influential in decision-making, even among well-intentioned providers (44).

Implicit biases, including unconscious attitudes and stereotypes, are prevalent among physicians, nurses, and other clinicians, and studies consistently show that the magnitude of these biases is similar to that found in the general population (45, 46). Hall et al. systematically reviewed 15 studies and found that healthcare professionals in the United States consistently demonstrated low to moderate levels of implicit pro-White or anti-minority bias, particularly against Black, Hispanic/Latino, and dark-skinned patients, as measured by the Implicit Association Test (IAT), a widely used reaction-time–based psychological measure that assesses automatic associations between social categories (e.g., race) and evaluative attributes (e.g., good or bad). Various versions of the IAT have been developed and used in research settings, including those available through Harvard’s Project Implicit platform (47). These levels of bias were comparable to those found in non-healthcare populations (45). Fitzgerald and Hurst reviewed 42 studies and similarly concluded that healthcare professionals exhibit implicit bias at rates equivalent to the broader population, with 35 studies finding evidence of implicit bias and all studies investigating correlations reporting a significant positive relationship between implicit bias and lower quality of care (4).

Evidence suggests that implicit bias in healthcare is not uniform across patient characteristics. Stigma toward individuals with mental health conditions may exceed biases related to race, weight, or socioeconomic status, and stigma toward patients with opioid use disorder may be even greater (48, 49). These findings highlight the complexity of bias in clinical practice and underscore the need to address multiple, intersecting forms of stigma within healthcare systems. Meidert et al. found that although racial bias was most frequently studied, gender, weight, socioeconomic status, and mental illness biases were also prevalent, especially among physicians and nurses in the United States (46). Research using the IAT and other measures confirms that implicit bias is widespread and can influence clinical decision-making, patient-provider interactions, treatment recommendations, and, ultimately, patient health outcomes (45, 50, 51). For instance, Hall et al. reported that implicit bias was significantly related to patient-provider interactions, treatment decisions, treatment adherence, and patient health outcomes, with the strongest associations observed in patient-provider interactions and health outcomes (45). Maina et al. found that all studies examining real-world patient-provider interactions reported that providers with more substantial implicit bias demonstrated poorer communication with patients (51).

## Implicit bias in pharmacy practice

Although research has primarily focused on physicians and nurses, growing evidence demonstrates that implicit bias is also prevalent among pharmacists and pharmacy students, influencing clinical interactions and patient care. Studies using the IAT indicate that pharmacy students often demonstrate negative biases toward Black patients, affecting assumptions about adherence and confidence in the provision of care (8, 15, 52). For example, Bunting et al. found that students assumed Black men would be less adherent to pre-exposure prophylaxis (PrEP), with higher implicit racism linked to lower confidence in PrEP-related care (15). Qualitative and cross-professional research further highlights that pharmacy students recognize a range of potential biases, including those related to race, ethnicity, insurance status, weight, and age. Yet, many of these students remain unaware of their own implicit attitudes and the attendant effects on behavior (5, 53). Broader studies also reveal consistent implicit preferences for White and light-skinned individuals across pharmacy, nursing, and medical education, reflecting the pervasiveness of these biases (52).

Among practicing pharmacists, implicit bias is increasingly recognized as a driver of health disparities. A 2024 study by Alzahrani et al., which surveyed 407 community pharmacists in Ontario, Canada, found a moderate implicit preference for White individuals over both Black and Arab people (54). Likewise, simulated patient studies demonstrate that pharmacists’ interpersonal responses can vary depending on patients’ perceived racial and ethnic backgrounds, even when objective service outcomes remain unchanged (7, 51). Collectively, this evidence suggests that implicit bias, whether among students or practicing pharmacists, can subtly undermine communication, trust, and equity in care, particularly in the management of chronic diseases, where pharmacists play a critical role in supporting adherence and long-term outcomes (8, 15, 53).

Implicit bias significantly impacts medication adherence through both direct and indirect pathways. When pharmacists harbor unconscious biases, it often manifests as poorer communication, reduced empathy, and a less collaborative approach during counseling sessions (51). These subtle cues can decrease patient trust and satisfaction, making patients less likely to follow complex therapeutic regimens or return for necessary follow-up care (45).

Furthermore, bias in clinical judgment may lead a practitioner to prematurely label certain groups as “non-adherent” based on stereotypes rather than investigating individual barriers. This labeling can result in a “self-fulfilling prophecy” where providers offer less intensive education or fewer adherence-support interventions to those they perceive as less likely to comply, ultimately widening health gaps in chronic disease management (6, 15).

## Evidence-based mitigation strategies

Growing evidence suggests that although implicit bias is deeply embedded in human cognition, it can be mitigated through cognitive unlearning and reassociation (43, 55). Rather than eliminating unconscious associations entirely, interventions focus on increasing awareness and interrupting automatic responses to promote more equitable decision-making (4). Studies, including randomized controlled trials and educational interventions, show that strategies such as perspective-taking, mindfulness, and bias-awareness training can reduce the influence of stereotypes on clinical judgment.

However, the sustainability of these effects often depends on the clinical environment. While individual-level interventions may produce short-term improvements, lasting change typically requires combining these strategies with organizational reforms, such as standardized protocols and bias-resistant workflows. Consequently, effective mitigation involves both individual awareness and supportive institutional systems, providing the foundation for the strategies outlined in the following sections (10, 37).

## Individual-level strategies

Pharmacists can begin by developing self-awareness and employing simple, in-the-moment techniques to manage and reassociate bias during patient interactions.

### Fostering self-awareness

The foundation of bias mitigation lies in recognizing one’s own susceptibility to unconscious bias. Structured self-reflection is the most effective way to cultivate this awareness and prevent automatic judgments from shaping professional behavior.

Regularly completing IATs, such as those developed through Harvard’s Project Implicit, can help individuals uncover hidden associations that may unconsciously influence their actions. The IAT is widely utilized in the literature as it provides a validated, objective measure of automatic associations that bypasses the limitations of self-reporting, where social desirability bias often masks implicit preferences (45, 56). While it is not a perfect predictor of individual clinical behavior, it remains a robust tool for identifying population-level trends and serves as a critical pedagogical “primer” to increase personal awareness of susceptibility to bias (4, 53).

While a specific standardized frequency for testing has not been established in the literature, evidence suggests that these tools are most effective when used as longitudinal indicators of automatic preference rather than one-time diagnostic tests (56). Practitioners should consider retaking the IAT periodically—such as during annual professional development or when entering high-pressure clinical environments—to maintain the mindful attention required to trigger corrective cognitive strategies (9).

These tools should not be viewed as diagnostic tests to identify prejudice but, rather, as indicators of automatic preferences that warrant mindful attention and corrective strategies (56). Reflective practice further strengthens self-awareness by encouraging individuals to analyze their own thoughts, emotions, and behaviors during challenging patient interactions. Keeping a reflective journal and asking questions such as, “Did I make assumptions about this patient?” and “Did I communicate differently with this patient than other patients today?” can reveal subtle bias patterns and guide behavioral improvement (57). Finally, peer discussion groups can support bias awareness by creating structured opportunities for health providers to reflect on challenging patient interactions and explore potential biases in a supportive environment. These groups may be implemented through facilitated case discussions, reflective practice meetings, or continuing professional development workshops, where participants share experiences, discuss bias-related scenarios, and collaboratively identify strategies for more equitable patient care. Such dialog helps normalize conversations about bias, promotes collective learning, and reinforces shared responsibility for equitable and respectful care (58).

### Active mitigation techniques

Awareness alone is insufficient. Instead, it must be paired with active, in-the-moment strategies to interrupt bias. The following evidence-based techniques are designed to help pharmacists deliberately engage in more conscious, patient-centered thought processes (Table 1).

**Table 1:** Evidence-based bias mitigation strategies.

Strategy	Description	Example
Individuation (88)	Focus on the patient’s unique history, not stereotypes.	Before an MTM session, actively review a patient’s specific history, notes, and preferences to better focus on the patient as an individual.
Perspective-taking (89)	See the encounter from the patient’s viewpoint.	Instead of labeling patients as ‘non-adherent,’ consider their unique perspectives: What financial or cultural barriers might they be facing?
Partnership building (88)	Promote collaboration and shared decisions.	Use collaborative language, such as, ““Let us review these options together to see what works best for your routine,” to create a shared plan.
Mindfulness (STOP) (89)	Pause and reset before acting by using the STOP acronym: Stop, Take a breath, Observe (thoughts/feelings), and Proceed with intention (89).	Before entering a challenging consultation, take a deep breath to pause, reset any automatic judgments, and proceed with intention.
Structured methodologies (69)	Use standardized tools and protocols to reduce subjective bias.	Use a standardized checklist during medication reconciliation to ensure every patient receives the same standard of thoroughness, regardless of background.
Stereotype replacement (55)	Replace biased thoughts with accurate, objective assessments.	Instead of assuming a patient understands, actively replace that assumption by using the teach-back method to confirm comprehension.

## Organizational strategies

Effective bias mitigation requires more than individual awareness. It must be embedded at the organizational level through standardized protocols that reduce subjectivity, a culture that promotes equity and inclusion, and workflows that integrate these practices into daily operations.

### Standardizing processes

Standardizing organizational processes is a key strategy for reducing subjective decision-making and minimizing the influence of individual biases. Implementing structured protocols, checklists, and data-driven decision tools promotes consistency and transparency in clinical workflows and patient care processes, thereby reducing the influence of individual bias (59, 60). In the context of AI-driven systems, ensuring algorithmic fairness is essential to prevent the automation of inequity. A critical challenge in this area is the impact of non-diverse or non-representative datasets, which often reflect existing societal and historical biases (61, 62). When machine learning models are trained on such data, they may produce skewed outcomes that are less accurate or even harmful for marginalized populations (63).

Mitigating these risks involves standardizing data collection to ensure inclusive representation and utilizing technical strategies—such as synthetic data augmentation or fairness-aware model training—to detect and correct bias at every stage (61, 63). Such systematic oversight helps prevent the reinforcement of existing disparities and promotes equitable outcomes in both human and automated decision-making (64, 65).

### Culture and well-being

Creating a culture that prioritizes diversity, equity, and inclusion (DEI) is essential for promoting fairness and psychological safety within pharmacy organizations. Leadership commitment, inclusive messaging, and the establishment of safe spaces for dialog help embed DEI values into everyday practice and decision-making (66, 67). Sustaining cultural change also requires clear accountability structures. Embedding responsibility for DEI objectives at all organizational levels ensures shared ownership of progress and prepares teams to address defensive reactions or resistance from dominant groups, fostering resilience and long-term commitment (66, 68). Ultimately, continuous feedback, reflection, and allyship are essential to sustain momentum. Encouraging open discussion and collective reflection on experiences reinforces bias mitigation as an ongoing, shared organizational value rather than a one-time initiative (67).

### Workflow integration

Directly embedding bias-resistant practices into organizational workflows promotes sustained equity and accountability. Bias-resistant practices refer to structured methods designed to minimize the influence of individual subjectivity by standardizing how information is processed and decisions are made (59). In pharmacy practice, this may involve implementing standardized clinical protocols, checklists, and decision-support tools to promote consistent patient care and minimize subjective decision-making (59, 64, 69).

Equally important is the establishment of continuous monitoring systems, which are persistent protocols used to evaluate the impact of workflows on equity over time. These systems involve regular audits of clinical outcomes and administrative decisions, as well as the evaluation of AI models using specific fairness metrics to detect “algorithmic drift” or emerging disparities (65, 70). This ongoing vigilance supports a dynamic, adaptive approach to equity within organizational practice (64, 65).

## Embedding equity in pharmacy education: curriculum reform and lifelong learning

Equity in pharmacy must be established through both foundational education and ongoing professional development. Research highlights persistent gaps in addressing health disparities, bias, and cultural humility within pharmacy curricula, as well as the need for continuous learning throughout pharmacists’ careers.

### PharmD curriculum reform

Given the already substantial demands of PharmD curricula, integrating topics related to implicit bias, health disparities, and cultural humility into existing courses may be more feasible than introducing entirely new standalone content. Embedding these concepts within established coursework, such as therapeutics, patient communication, public health, and experiential education, allows programs to reinforce principles of equitable care without substantially increasing curricular burden.

PharmD curriculum reform should therefore focus on systematically incorporating these topics across multiple stages of pharmacy education. Effective approaches include case-based learning, community engagement activities, and interprofessional education experiences that expose students to diverse patient populations and social determinants of health (71–73). Integrating these themes longitudinally throughout the curriculum helps reinforce their relevance to clinical decision-making and professional practice, promoting meaningful and sustained learning (74, 75).

Addressing the hidden curriculum is also essential. The hidden curriculum refers to the informal lessons, values, and norms conveyed through the educational environment and institutional culture (76). For example, teaching materials that consistently associate certain populations with non-adherence or clinical complexity may unintentionally reinforce stereotypes among learners (77). Reviewing course materials, case studies, and assessments to minimize implicit bias and promote equitable representations of patients can help mitigate these unintended influences (76, 77).

Finally, fostering diversity among faculty and students can further support inclusive learning environments. Diverse perspectives enrich classroom discussions, enhance cultural understanding, and help prepare future pharmacists to deliver patient-centered care in increasingly diverse communities. Recruitment, retention, and mentorship initiatives that support diversity within pharmacy education can therefore play an important role in advancing equity within the profession (78, 79).

### Lifelong professional development

Pharmacy education and practice should embrace lifelong professional development grounded in cultural humility. This approach moves beyond cultural competency to emphasize continuous self-reflection, openness, and responsiveness to the diverse needs of patients. Evidence demonstrates that ongoing education and interactive training improve practitioners’ understanding and confidence in addressing bias and health disparities (80–82). Licensing boards and professional organizations should mandate continuing education in equity, diversity, and bias mitigation as a core component of professional growth. Requiring such training ensures that equity principles remain integral to pharmacy practice throughout a pharmacist’s professional career (78, 80). Furthermore, an equity lens should be applied throughout pharmacists’ patient care process. Considering social determinants and structural barriers at every step—from assessment to follow-up—helps ensure that care decisions are equitable, patient-centered, and culturally informed (73, 83).

## Limitations and future research

Despite progress in understanding and addressing implicit bias in pharmacy, several methodological and practical gaps persist, limiting the field’s ability to guide effective practice and policy.

### Methodological limitations

Pharmacy bias research faces several methodological challenges that limit the strength and generalizability of its findings. Many studies rely on small, cross-sectional, or single-site designs, which restrict the ability to draw broad conclusions across diverse settings (84, 85). Additionally, there is a dearth of large-scale, multi-site, randomized controlled trials that are capable of rigorously evaluating the effectiveness of bias-reduction interventions (86). Another limitation lies in measurement tools. Most studies depend on generic instruments such as the IAT, which may not fully capture pharmacy-specific contexts or observable behaviors. Future research should prioritize developing and validating tools tailored to pharmacy practice and should also include methods that assess real-world actions, not merely implicit attitudes (84, 87). Furthermore, few extant studies address intersectionality—how overlapping social identities such as race, gender, socioeconomic status, and disability interact to shape bias and patient care experiences. Ignoring these intersecting factors risks oversimplifying bias and missing critical nuances in both provider behavior and patient outcomes (87).

### Intervention and outcome gaps

A lack of long-term and patient-centered outcome data limits current research on bias mitigation in pharmacy practice. Most interventions measure short-term changes in implicit or explicit bias scores without examining their impact on meaningful outcomes, such as patient satisfaction, medication adherence, and clinical results (86, 87). Evaluating whether bias reduction efforts translate into improved patient care and health equity remains a crucial next step. Moreover, the cost-effectiveness of bias mitigation strategies is rarely assessed. Understanding the financial implications and return on investment of these interventions is essential in guiding institutional policies and resource allocation. Without such evidence, organizations may struggle to justify sustained implementation of equity-focused programs (86).

### Innovation and technology

Emerging technologies offer promising avenues to address bias in pharmacy education and practice. Artificial intelligence (AI) and data science can be leveraged to audit prescribing patterns, identify disparities, and support equitable decision-making. Similarly, virtual and augmented reality tools provide immersive training environments that enhance awareness and empathy by simulating diverse patient experiences. Despite their potential, these innovations remain largely underexplored within pharmacy bias research (61–63). However, several technical limitations hinder widespread adoption. Many AI-based bias mitigation solutions lack standardization, face challenges in integration with existing systems, and are difficult to implement in real-world clinical environments. Moreover, current algorithms often fail to capture the full complexity of bias across diverse populations, highlighting the need for more inclusive data sets and ethically guided technological design (61, 62).

## Conclusion

Implicit bias remains a significant factor in pharmacy practice, contributing to disparities in communication, clinical decision-making, and patient outcomes. Although these biases arise from deeply ingrained cognitive processes, evidence suggests that their effects can be mitigated through targeted interventions. Combining individual strategies, such as awareness, reflection, and cognitive techniques, with organizational approaches, including standardized protocols and supportive practice environments, may help reduce the influence of bias in clinical care.

Integrating these principles into pharmacy education and ongoing professional development is essential to prepare pharmacists to deliver equitable, patient-centered care. However, further research is needed to evaluate the long-term effectiveness of bias mitigation strategies and their impact on patient outcomes. Continued commitment from educators, practitioners, and professional organizations will be critical to advancing health equity and improving medication-related outcomes for diverse patient populations.
